# Implementing pharmacogenetic-guided prescribing in general practice: a qualitative process evaluation

**DOI:** 10.1186/s43058-026-01005-x

**Published:** 2026-06-26

**Authors:** Lisa Brunton, William Newman, John McDermott, Paul Wilson

**Affiliations:** 1 Centre for Primary Care and Health Services Research, Division of Population Health, Health Services Research and Primary Care, School of Health Sciences, University of Manchester, Williamson Building, Oxford Road, Manchester, M13 9PL UK; 2 Division of Evolution, Infection, and Genomics, School of Biological Sciences, University of Manchester, Manchester, UK; 3 Manchester Centre for Genomic Medicine, Manchester University NHS Foundation Trust, Manchester, UK

## Abstract

**Background:**

Some people experience side-effects from medicines which can lead to negative patient outcomes and increased NHS costs. Pharmacogenetic-guided prescribing supports the safety and effectiveness of medicines. The Pharmacogenetics Roll Out – Gauging Response to Service (PROGRESS) study is assessing the viability and utility of pharmacogenetic-guided prescribing in general practice. We conducted a parallel process evaluation to explore the barriers and enablers to implementing pharmacogenetic-guided prescribing in general practice, from a clinician perspective.

**Methods:**

Twenty-nine online semi-structured interviews were conducted with 30 general practice staff across 20 sites in England implementing the PROGRESS study. We used a modified framework approach to analyse the data, informed by the Consolidated Framework for Implementation Research and Normalisation Process Theory.

**Findings:**

Clinicians saw value in offering pharmacogenetic-guided prescribing in general practice, but limited testing to where they perceived it provided greatest benefit. There was limited evidence of practices planning for implementation beyond setting up the PROGRESS study. Sites tailored delivery of pharmacogenetics to their local practice context, which acted as an enabler to implementation. Delivering pharmacogenetic-guided prescribing within the confines of a research study and clinicians’ workload acted as barriers to implementation.

**Conclusion:**

General practice staff understood the value and purpose of pharmacogenetic-guided prescribing, but there was little evidence of wider practice involvement beyond raising awareness among staff. PROGRESS involvement was viewed as more as proof of concept for research purposes, rather than normalising pharmacogenetics into routine practice.

**Supplementary Information:**

The online version contains supplementary material available at 10.1186/s43058-026-01005-x.

Contributions to the literature • Our study shows clinicians’ receptiveness to the value of pharmacogenetic-guided prescribing and enabling local level adaption as key enablers to implementation.

• Key barriers to implementation included current workload pressures, and pharmacogenetic-guided prescribing being implemented within the restrictions of a research study, limiting wider clinician engagement across sites.

• From our findings, we make recommendations for future scale-up of pharmacogenetic-guided prescribing across general practice in the UK. These include the need to improve communication and feedback within practices, ensure greater multidisciplinary team involvement, improve clinical education and guidance, and ensure sufficient resources (including integrating pharmacogenetic test results into practice records).

## Introduction

Medicines are the most common intervention in healthcare, with the NHS prescribing around 1.1 billion items each year [1]. Medicines play a significant role in preserving health, preventing illness, managing long term conditions and treating diseases [2]. However, variability in drug response can mean that some people experience reduced medicines effectiveness or develop side effects. This can lead to poorer patient outcomes and increased healthcare costs; for example, adverse drug reactions are projected to cost the NHS in England £2.2 billion per year [3].

Genomic factors contribute to inter-individual variability in drug response, a concept known as pharmacogenetics. Using pharmacogenetics in clinical practice to guide prescribing can support improvements in medicine safety and effectiveness [3, 4]. Evidence-based guidelines, such as those published by the Clinical Pharmacogenetics Implementation Consortium (CPIC), are available to support pharmacogenetic-guided prescribing for commonly prescribed medications [5]. Yet, despite robust evidence, implementation of pharmacogenetics into clinical practice has remained slow [4]. Recent policy initiatives support the implementation of genomic medicine into UK health care [6–8], and the Royal College of Physicians and the British Pharmacological Society have produced a report with key recommendations for implementation of pharmacogenetics in the UK NHS [9].

Evidence-based guidance recommendations on their own are not enough to affect change. Implementation science (the study of methods to promote the uptake of evidence-based interventions) can provide insights to enhance the integration of pharmacogenetics into real-world practice. However, some evidence-based practices depend on technical infrastructure, decision support systems and regulatory arrangements all being in place before implementation can be evaluated. Pharmacogenetics is one such “implementation object” where, in the context of implementation science, the required changes in delivery structures may be conceptualized as implementation strategies which can influence the uptake of the “implementation object” into practice [10]. Using an implementation science lens can identify the necessary changes in delivery structures that influence successful implementation, it can provide insights on whether the “implementation object” can actually be delivered in the real-world settings, and facilitate a shared understanding of what is required to routinise pharmacogenetics in everyday practice [11].

**Fig. 1:**
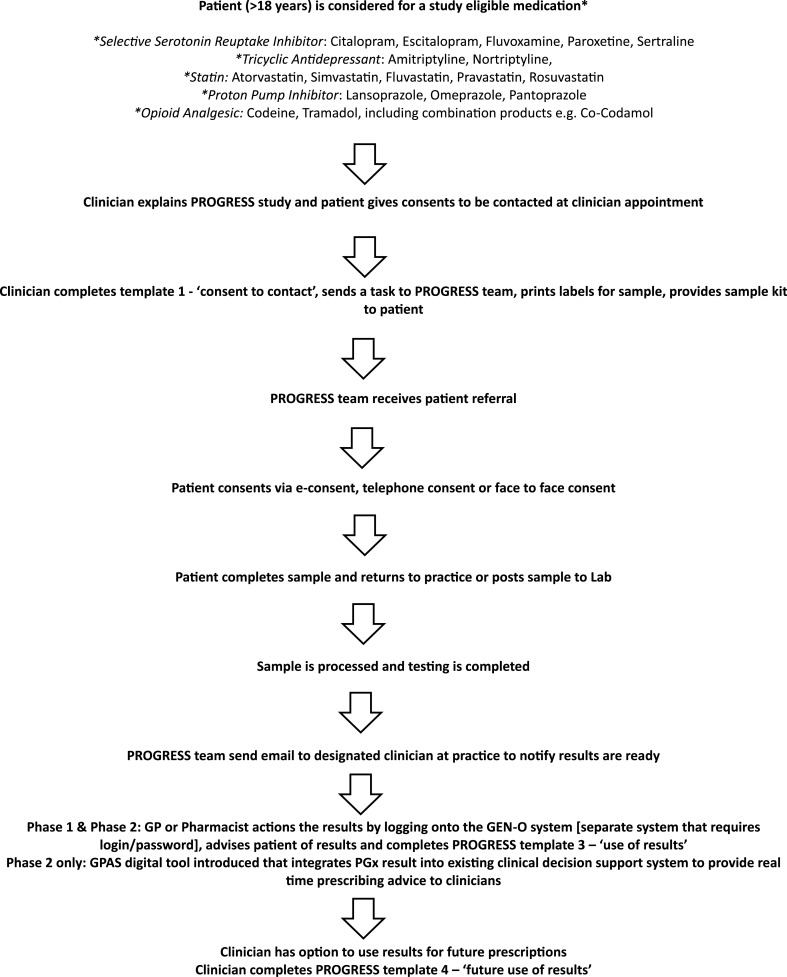
Steps involved in PROGRESS pharmacogenetics intervention

It is argued that the model for pharmacogenetics implementation needs to account for the need for ongoing learning and evidence development [11]. The Pharmacogenetics Roll Out – Gauging Response to Service (PROGRESS) study (ISRCTN15390784) is part of a programme of work which aims to do just this by introducing evidence-based pharmacogenetic-guided prescribing in general practice in England, UK [12]. The PROGRESS study is a pragmatic, observational implementation study which aims to assess the viability and utility of pharmacogenetic-guided prescribing in general practice (see Fig. 1). This requires the implementation of an end-to-end pathway, with mechanisms for patient consenting, test ordering, sample transport, laboratory testing, and return of results to guide prescribing practice. The study is set in UK general practice, where GPs and allied health professionals deliver first-contact care within the publicly funded NHS. General practice was chosen as the setting as most prescriptions are initiated there [12], with general practice appointments accounting for almost 60% of all patient contact across the NHS [13]. In addition, the infrastructure exists primarily to support medicines optimisation and prescribing in this setting (for example, general practice clinicians use clinical decision support systems to consider co-morbidities and current medications when prescribing) [14]. PROGRESS was conducted in two distinct phases, enabling an iterative approach to testing, learning and scaling up implementation across England. In phase 1, PROGRESS was implemented in 10 early adopter sites across the Northwest of England, from June 2023. This phase focused on initial roll out and evaluation to test the feasibility and acceptability among staff and enabled a refinement of implementation processes before wider roll out in a further 11 sites (phase 2 sites), from February 2025, across England, to broaden adoption and assess scalability and impact across more diverse settings. A key innovation following phase 1 was the Genomic Prescribing Advisory System, known as PROGRESSRx, a digital tool that integrates genetic results into existing clinical decision support systems, to provide real-time prescribing advice to general practice clinicians.

Alongside the PROGRESS study, we carried out a process evaluation, utilising implementation science methodology. This study aims to shed light on the structures, resources and processes through which successful delivery of pharmacogenetic-guided prescribing into general practice in England can be achieved.

## Methods

We report our methods using the Consolidated Criteria for Reporting Qualitative Studies (COREQ) checklist for qualitative research [15].

### Study design and setting

We undertook qualitative, semi-structured interviews, with staff across 20 out of the 21 general practice sites implementing PROGRESS, across England. The rationale for a semi-structured interview approach was that it would enable us to gather insights from those directly involved in or affected by the introduction of pharmacogenetic-guided prescribing. This allowed in-depth exploration of the context, including workflow implications, organisational change, and any potential barriers to implementation.

### Theoretical approach

We drew upon two theories to explore what is needed to support the implementation of pharmacogenetic-guided prescribing in to general practice: Normalisation Process Theory (NPT) [16, 17] and the Consolidated Framework for Implementation Research (CFIR) [18]. NPT is an implementation process theory to explain the work that people do to implement new innovations into routine practice and how the work is done [16] while CFIR describes the contextual factors of implementation [18]. These two theories have been successfully combined in previous studies to inform different aspects of implementation [19, 20]. Both theories informed the development of a topic guide, used for data collection, and analysis.

### Participants and recruitment

We aimed to recruit a purposive sample of different staff roles involved in the implementation and delivery of pharmacogenetic-guided prescribing at the general practice level. At each site, a key contact was identified; these individuals had primary responsibility for overseeing implementation of PROGRESS at their practice. We allowed at least three months from them starting PROGRESS before we recruited participants. The key contact was sent a pre-prepared email with a participant information sheet to invite them to take part. After interviewing the key contact, we undertook snowball sampling [21], whereby a pre-prepared email invite and participant information sheet was forwarded to other staff members identified by the key contact as being involved in delivering pharmacogenetic-guided prescribing at their practice. After two weeks, a reminder email was sent to potential participants if we received no reply.

### Data collection

Semi-structured interviews were conducted by LB, a female post-doctoral research associate, with extensive experience in qualitative research methods and implementation science. LB had no prior professional or personal relationship with any of the study participants. All interviews were carried out via Microsoft Teams and audio recorded with participants’ consent. Interviews were undertaken between May 2024 and October 2025 and the mean duration of the interviews was 45 min.

We developed a theoretically informed topic guide (see supplementary file 1 - topic guide) to explore the barriers and enablers to integrating pharmacogenetic-guided prescribing in general practice, and to explore the views and experiences of those delivering PROGRESS.

We offered financial reimbursement to general practices to compensate for staff time participating in the study. Payments were aligned with the National Institute for Health and Care Research guidance on research cost attribution and were based on standard reimbursement rates at the time of the study (£102 per GP interview and £44 per allied health professional interview).

### Data analysis

Interviews were transcribed. Each transcript was checked for accuracy and identifying information removed by LB. An interview summary was created for each transcript. Transcripts were uploaded to NVivo 14, a software package for qualitative data [22]. Transcripts were first coded by LB, using inductive coding [23] (see supplementary file 2 - coding tree). Initial codes were refined by LB and PW, using a modified framework approach [24] to produce a matrix of summarised data creating a structure for further analysis. Subsequent theme refinement was undertaken by all authors and guided by CFIR and NPT. This enabled consideration of the specific research questions, while also allowing inductive insights.

## Results

Thirty participants from 20 sites took part in the study. Eighteen interviews were carried out with 19 participants from *n* = 10 phase 1 sites, as one interview was carried out with two participants (a GP and a practice manager). Eleven interviews were carried out with participants from *n* = 10 phase 2 sites. Table 1 shows the distribution of participant interviews across phase 1 and phase 2 sites and participants’ work roles.

**Table 1:** Explanation of interviews conducted across sites and participants’ professional roles

**Practice ID**	Interview number	Professional role/s *Indicates was local PI **indicates was associate local PI
**Phase 1 sites**		
Practice 01	1	GP*
Practice 02	2	GP* and Practice manager interviewed together
Practice 03	3	GP*
	4	GP trainee
Practice 04	5	GP*
	6	GP trainee
	7	Clinical pharmacist
	9	GP
Practice 05	8	Academic GP*
	10	GP
	12	GP
Practice 06	11	Clinical pharmacist*
Practice 07	13	GP*
	14	Clinical pharmacist
	16	Research nurse
Practice 08	15	GP*
Practice 09	17	GP*
Practice 10	18	Clinical and research pharmacist*
**Phase 2 sites**		
Practice 11	19	GP*
Practice 16	20	GP*
Practice 12	21	Clinical pharmacist*
Practice 13	22	Clinical pharmacist*
	28	Clinical pharmacist**
Practice 14	23	GP*
Practices 15 & 20	24	GP*
	26	Research Nurse
Practice 17	25	Pharmacist*
Practice 18	27	Pharmacist*
Practice 19	29	Pharmacist*

Results are presented across four overarching themes derived from the data, offering insights into factors influencing implementation of pharmacogenetic-guided prescribing across phase 1 and phase 2 sites. Table 2 provides a selection of direct quotes to support the interpretation of findings, while supplementary file 3 provides a more comprehensive compilation of quotes.

**Table 2:** Quotes to support findings

Theme	Quote
1. Perceived value influences prescribing practices	And I think if it was just in your arsenal, it would really alter the way that you communicate with this stuff… we’re moving from paternalism into shared decision making, aren’t we? Interview 13, GP (local PI), practice 07, phase 1 site. I think it has definitely changed how I approach statins, I think, in terms of… the ones who are most resistant to it. Interview 24, GP (local PI) practices 15 & 20, phase 2 sites. …to be honest, I’d mostly use it for SSRIs […] I guess from my personal experience, they’re the ones that tend to be hit and miss and people chop and change on [SSRIs] a lot more, so to, like, try and negate some of that. Interview 4, GP trainee, practice 03, phase 1 site.
2. Planning for implementation?	But what was quite good was they were still, sort of, codesigning the project over the time we were speaking to them…And so, we were involved maybe, you know, in helping shape that to a degree and helping advise [PROGRESS study team]. So, I suppose that helped to allay some fears, that you’re an active participant in that. Interview 1, GP (local PI), practice 01, phase 1 site. And I’m actually going to give them feedback on the first hundred patients at a teaching session in September…so, I think certainly, like, upskilling primary care clinicians in this is…was one goal. Interview 21, Pharmacist (local PI), practice 12, phase 2 site. So, I got so far as to, you know, even recording my own short videos, apart from sharing all the training material information, recorded some two-minute knowledge nudges and things like that and shared them through WhatsApp…. Interview 17, GP (local PI), practice 09, phase 1 site.
3. Delivering pharmacogenomics in practice: tailoring to local context	…it would certainly be on judgement, and it would be on that scenario and that particular patient […] And if I felt that it was necessary to start that medication first, then I would, I wouldn’t hold off. Interview 18, Pharmacist (local PI), phase 1 site. I always prescribe and then amend. So, I don’t do both. And I think everyone in our practice does that. So, I think the decision was don’t alter the [current prescribing practice]. Interview 9. GP, practice 04, phase 1 site. Some of it comes down to, what’s your attitude to your responsibility with cost-saving within the NHS? You’ve prescribed something and it’s the incorrect thing, then it’s a wasted prescription, wasted medication, it’s wasted pharmacy times and stuff. Interview 3, GP (local PI), practice 03, phase 1 site.
4. Delivering pharmacogenomics in practice: barriers to use	A couple of the salaried [GPs] said, if I’ve got a late surgery, I cannot be expected to do this. And that was the end of that conversation. Interview 16, Research Nurse, practice 07, phase 1 site. So in my practice, and because we’re newly setting up research, I’m the only one who is GCP trained and can actually do it, I’m the only person in the practice who is doing it. Interview 29, Pharmacist (local PI), practice 19, phase 2 site. …trying to convince other doctors especially to start talking about this, et cetera, and take the lead on it and do all the stuff, I think it’s a bit much for them. Interview 23, GP (local PI), practice 14, phase 2 site.

### Perceived value influences prescribing practices

Most respondents perceived pharmacogenetic-guided prescribing to be a relative advantage to current prescribing pathways, with some stating it was the *‘future’* of prescribing. Respondents recognised the benefits of using pharmacogenetic-guided prescribing in general practice: being able to personalise or tailor medications to patients, preventing harm by reducing the risk of prescribing the wrong medications, and having test results available to inform future prescribing. Respondents also perceived that it had the potential to increase efficiencies, by ensuring that the right medication was prescribed first time. Some respondents felt that pharmacogenetic-guided prescribing helped shape conversations with patients, shifting them away from a paternalistic approach and towards shared decision-making.

Respondents’ views of where pharmacogenetics offered greatest clinical benefit influenced how they delivered the test in practice. Several reported pharmacogenetic testing provided benefit when prescribing statins and/or anti-depressant medications, with some indicating only offering it when prescribing these medications. When prescribing statins, negative media reporting surrounding side effects was believed to increase patients’ hesitancy to starting statins. They felt pharmacogenetic test results could increase medication adherence by reassuring patients that the correct statin was being prescribed.

When respondents perceived pharmacogenetic testing offered greatest benefit for anti-depressant medication prescribing, they reported patients having tried several medications before finding one suitable, and therefore perceived value in offering pharmacogenetic testing to mitigate this.

Some respondents perceived the test had less utility and reported not using pharmacogenetic testing when prescribing PPIs and/or opiates. Some felt prescribing decisions for PPIs were less complicated, preferring to base their decision on patients’ symptomatic relief and/or side-effects experienced. Others reported less utility in offering the test when co-prescribing PPIs for short courses of anti-inflammatory medications: *I…feel like*,* it is hardly worth it* (Interview 9 (GP) phase 1 site).

When respondents perceived less utility for pharmacogenetics in opiate prescribing, reasons were twofold: some held concerns that prescribing options for chronic pain were already limited and test results could lessen their prescribing options further, while others reported a drive to reduce opiate prescribing at their practice, lessening the need for pharmacogenetic testing.

### Planning for implementation?

Some local PIs worked with the PROGRESS study team in early study intervention design, while others collaborated to test how the results would integrate with PROGRESS Rx workflows. These collaborations helped build a shared understanding of the intervention, clarifying how it would align with existing practices, and resolving early technology issues.

At a practice level, while there was evidence of local PIs planning for implementation in terms of anticipating barriers to implementation, there was less evidence of cognitive participation or collective action where the wider practice team would come together to set implementation goals, develop implementation plans, or monitor implementation. In most cases, efforts were primarily focused on setting up the PROGRESS study. However, two sites in phase 2 (practice 11 and practice 12) took a proactive approach by independently monitoring their data and planned to present their local evaluation findings more formally to staff, with the aim of increasing awareness and fostering greater engagement across their practices.

In addition, local PIs across both phase 1 and phase 2 sites, in planning for implementation, did anticipate barriers to use: clinicians forgetting to offer the test during consultations or not offering the test due to feeling overburdened during consultations. To address these barriers, local PIs created tools to aid delivery. These included creating ‘pop-ups’ on practice systems to alert prescribers to offer pharmacogenetic testing to their eligible patients, creating laminated documents explaining the recruitment process (including step-by-step guides to completing templates), and creating ‘live demonstration’ videos of completing templates. There was some evidence of networking across sites to support implementation planning, such as sharing of resources (e.g. pop-ups) and addressing early implementation issues.

Ahead of implementation, local PIs and other practice research team members engaged with key practice staff to explain the benefits of pharmacogenetic-guided prescribing and train them in the process of recruitment. Engagement took the form of face-to-face presentations during regular practice meetings or more formal training sessions. Some combined these efforts with other strategies including sending out relevant information via their dedicated practice social media channels.

### Delivering pharmacogenetics in practice: tailoring to local context

The PROGRESS study team enabled practices to tailor implementation of the intervention to fit their local context and there was evidence of practices adapting the intervention to fit their needs. Specifically, this related to differences identified when practices started patients’ medications (i.e., before or after test results were returned), differences in how test results were organised and processed, differences in how patients were recruited and how some phase 2 practices used pharmacogenetic results to guide prescribing beyond study medicines.

Across phase 1 and phase 2 sites, most respondents based their decision of when to prescribe medications on the severity of patients’ symptoms, the nature of their complaint and/or the types of medications being prescribed. A small number of respondents reported always prescribing medications before test results were received, rationalising this fit with current prescribing practices, as they could not rely on clinicians being available to prescribe medications when test results were returned several days later. Others reported not wanting to delay prescribing medications for which there was a clinical need. By contrast, two respondents (phase 1 sites) reported prescribing medications only after test results were returned. One participant (interviewee 1) was an early adopter of PROGRESS and had learnt over time that it was better to prescribe medications after test results were returned to ensure the right medication was prescribed first time. Interviewee 3 considered it *‘illogical’* to start medications before receiving test results, as this risked wasting NHS resources if medications needed to be changed.

Practices also differed in how they managed test results, with most local PIs, across both phase 1 and phase 2 sites, taking sole responsibility. Local PIs were mindful not to overburden GPs with this extra workload. However, they acknowledged the burden placed on themselves to undertake this work, recognising it was only feasible due to the relatively small number of patients being recruited. One practice (site 4) had delegated the task of processing test results to their PCN practice pharmacists, to limit GP workloads. In phase 2 sites, several local PIs were pharmacists and considered themselves suitably qualified to interpret the findings. However, most respondents indicated that for pharmacogenetic-guided prescribing to be effectively integrated into routine practice, results should be incorporated into practice IT systems (as happened in phase 2 with the introduction of PROGRESSRx) and clinical staff would require upskilling in pharmacogenetics to facilitate interpretation. In addition, one participant (interviewee 25) recommended developing local, user-friendly guidelines because existing resources, such as the CPIC guidelines, were perceived to be overly technical.

Most sites across phase 1 and phase 2 recruited patients into PROGRESS opportunistically during consultations. However, a small number of phase 2 sites supplemented this with population health management approaches. For example, some conducted searches of patient records to identify individuals with elevated cardiovascular risk who were eligible for a statin but had not yet been prescribed one. These sites proactively invited patients to participate in PROGRESS when discussing statin therapy. This proved particularly successful in one site (practice 12), who had recruited almost 100 participants in the first four-months of introducing PROGRESS into their practice. In addition, one phase 2 site (practice 13) also attempted to implement a weekly search to identify eligible patients who were not offered PROGRESS during consultations that week, to invite them to take part. However, competing workload pressures limited adoption into routine practice.

### Delivering pharmacogenetics in practice: barriers to use

Pharmacogenetic-guided prescribing was not considered to be normalised into routine practice in any of the phase 1 and phase 2 sites. Most local PIs reported that only small numbers of enthusiastic clinicians within practices were offering pharmacogenetic-guided prescribing to their patients. Many respondents reported that high workload was a significant barrier to clinicians engaging with pharmacogenetic-guided prescribing. Time constraints and cognitive overload were identified as key barriers to offering the test opportunistically during consultations, as it added an additional task within a consultation. For future provision, respondents felt that pharmacogenetic testing needed to be appropriately commissioned and resourced, otherwise it was not considered sustainable.

In phase 1 sites, challenges were noted regarding the cumbersome referral and results processes, with some clinicians perceiving pharmacogenetic-guided prescribing as incompatible with existing workflows. These issues largely arose from implementing pharmacogenetic-guided prescribing within the confines of a research study, which required additional templates and tasks to refer patients to the research team for consent.

By contrast, phase 2 respondents reported that completing study templates was straightforward and commended the PROGRESS central team for simplifying these processes. However, despite these iterative improvements, integration into routine practice remained limited. One reason may be that local PIs, aiming to protect clinicians from additional workload, limited clinician involvement to simply introducing the study to eligible patients. This meant that clinicians did not get involved in the study’s processes or clinical implications of test results. This, together with the lack of feedback on study progress, as outlined above, may have contributed to lack of clinician engagement and lower recruitment levels across sites. Despite this, most respondents felt that general practice was best placed to offer pharmacogenetic testing in future and considered that burdens would be removed if the test was offered outside of a research study.

## Discussion

### Summary

This study revealed that general practice clinicians see value in offering pharmacogenetic-guided prescribing. However, despite its wider utility, there was evidence that clinicians limit pharmacogenetics to testing for certain medications, according to where they perceive it offers greatest clinical benefit. An enabler to implementation was the degree of plasticity; that is, the extent to which implementation of the intervention could be tailored to fit the local context [17], with sites adapting PROGRESS to their practice needs. However, pharmacogenetics was not considered to be embedded into routine practice across any of the 20 sites. The main barriers to this included the burden of the additional procedures required to deliver pharmacogenetic-guided prescribing within a research study context. As a result, responsibility for implementation often remained with local PIs and research team members which may have impeded broader clinician engagement. In addition, high workload and time pressures within consultations posed additional challenges.

Using CFIR [18] and NPT [16, 17] as our implementation science lens, helped to reveal the enablers and barriers to pharmacogenetic testing being embedded into routine practice. Using CFIR helped us to identify that although clinicians shared receptivity and alignment regarding the value of pharmacogenetics, there was reduced absorptive capacity for change (the ability to recognise, understand and exploit new knowledge). The changes required in delivery structures within available resources limited the full uptake of the “implementation object” across sites. Use of NPT helped identify that implementation was very much grounded in ‘coherence’ (or sense-making); understanding the purpose and value of PROGRESS and the work needed to enact and embed it into existing practice. There was little evidence of ‘cognitive participation’ and ‘collective action’ suggesting that practice involvement in pharmacogenetic-guided prescribing was viewed more as proof of concept for research purposes rather than the integration of a new way of working into existing practice. Establishing and building coherence so that practices move to action will be an essential prerequisite for routine integration of pharmacogenetics into clinical practice.

### Strengths and limitations

A strength of the study was that findings from phase 1 interviews were iteratively integrated into PROGRESS as further sites in phase 2 were onboarded across England. Feedback from interviews supported the development of PROGRESSRx. This highlights the value of qualitative assessments in the early phases of implementation studies to optimise processes.

We aimed to recruit a maximum variation sample, to elicit a wide range of views and experiences from people implementing and delivering PROGRESS across the 21 practices and were successful in recruiting respondents in a variety of different general practice staff roles. We encouraged key contacts to identify staff at their site who were involved in PROGRESS. This led to one practice manager being recruited; however, their involvement in PROGRESS was limited to assisting with study set up and prompting the GP to act on test results once they were received. Given the limited nature of their participation, this individual represents an outlier within our sample.

Our sample included representation from across 20 out of the 21 PROGRESS study sites; however, in five of the phase 1 sites, and in seven of the phase 2 sites, we managed only to recruit one respondent (the local PI), perhaps reflecting a lack of engagement from other clinicians to the PROGRESS study within practices. The study achieved a level of thematic saturation, as interviews yielded consistent and repeated themes and no new concepts emerged in later interviews. However, it may be that some eligible participants chose not to participate and therefore the findings may not fully represent the range of views and this should be considered when interpreting the results.

The findings of this study are grounded in the UK NHS context, where GPs play a central role in prescribing medications. As such, aspects of pharmacogenetic-guided prescribing identified here may be relevant to other healthcare systems with strong general practice infrastructures. However, transferability may be limited in settings where prescribing responsibilities are more fragmented, where access to pharmacogenetic tests are organised differently, or where healthcare is delivered through insurance-based models.

### Comparison with existing literature

Our research aligns with previous studies that explored general practice staffs’ experiences and attitudes to introducing pharmacogenetic testing into general practice [25, 26]. A key theme from a qualitative interview study conducted by Lemke and colleagues identified the perceived value and utility of delivering pharmacogenetic testing in general practice, with physicians highlighting benefits such as stopping side effects, ability to titrate doses, improving shared decision-making and providing reassurance to patients [26]. In a focus group study with general practice clinicians and patients, Frigon et al., identified enthusiasm for implementing pharmacogenetic testing into general practice, with most participants highlighting reduction of adverse events as the main benefit [25]. Our research, and others therefore highlight the *acceptability* of introducing pharmacogenetic testing into general practice; a key outcome necessary for implementation success [27].

We identified that practices tailored PROGRESS to fit their local context, and this acted as an enabler to implementation. Previous literature has identified that local level adaptation (i.e., the interaction between the intervention, the process of implementing the intervention, and the context in which it is implemented), can improve the fit and effectiveness of interventions and support sustainability [28–30].

A key barrier to implementation in the present study was the current workload pressures, leading to some clinicians being reluctant to take on the extra burden of introducing pharmacogenetic testing into their consultations. A recent report by the Royal College of General Practitioners highlights a service under strain, with significant ‘outer setting’ factors such as the increasing population demand for appointments and decreasing numbers of full-time equivalent GPs, adding to workload pressures [31]. This highlights the need to consider the context of general practices when introducing new services into this setting.

### Implications for practice

Findings from this study are timely, given the UK Government’s recently published the policy paper: Fit for the Future: 10-year Health Plan for England [8], which commits to integrating pharmacogenetic testing into the NHS over 40 Health Checks, starting in 2026. We offer the following implications for practice:

1. Communication and feedback: practices should establish clear processes to monitor who within the practice is offering the test and provide feedback to improve uptake and consistency.
2. Multidisciplinary team involvement: involving pharmacists in managing test results may enhance sustainability and reduce the burden on individual clinicians.
3. Clinical education and guidance: robust guidance is needed on when when pharmacogenetic testing is recommended. Clinicians need clearer guidance on when they should prescribe medications (before or after pharmacogenetic test results are available) and clinicians require upskilling to confidently interpret those results.
4. Integration with general practice records: long-term implementation will require sufficient resource and integration of pharmacogenetic test results into general practice records.

## Conclusions

Findings from this study highlight the feasibility of implementing pharmacogenetic testing in general practice in a UK context, but barriers need to be addressed for widespread uptake. Notably, clinicians were found to selectively offer pharmacogenetic tests to patients based on the perceived value of testing for a particular medication rather than strictly on patient eligibility. This clinician-based decision-making, based on personal clinical experience, highlights the need for more robust guidance on when testing is recommended. Additional barriers to be addressed include more robust planning ahead of innovation launch (to further engage clinicians beyond research teams), provision of sufficient resource, and streamlining processes for obtaining and organising results.

## Supplementary Information

Below is the link to the electronic supplementary material.

Supplementary Material 1

Supplementary Material 2

Supplementary Material 3

## Data Availability

The datasets generated and analysed during this study are available from the corresponding author upon reasonable request.
