# Supplementary material for: Implementing pharmacogenetic-guided prescribing in general practice: a qualitative process evaluation
Source: Implement Sci Commun. 2026 Jun 26;7:149. doi: 10.1186/s43058-026-01005-x (PMC13471391; doi:10.1186/s43058-026-01005-x)
Supplement: Supplementary file 1 [file 43058_2026_1005_MOESM1_ESM.pdf]

Process evaluation of PROGRESS (Pharmacogenetics Roll Out – Gauging Response to Service)
Interview topic guide (Health Professionals)

Ahead of interview starting:

- Reminder of the purpose of study
- Explanation of interview consent and confidentiality of interview and analysis
- Structure of the interview (may be some overlap in questions and responses)
- Can stop the interview at any point if you do not wish to continue
- Any questions you would like to ask before we start?
- Audio Recorder switched on

**Topics**	**NPT construct**	**CFIR domain**
**Professional role**<br>• Ask them to explain their current role<br>• How long have they worked there?<br>• Do they work in a research active/training general practice?	N/A	N/A
**Initial thoughts**<br>• Ask how they first heard about PROGRESS<br>• What did they think of PROGRESS when they first heard about it?<br>• How was the decision made to adopt and make it work in their setting? How did they participate in those decisions? What influenced their decision to participate? What did they hope to achieve?<br>• Ask what they liked/did not like about the idea of using PROGRESS	Coherence, Cognitive Participation and Reflexive Monitoring	Intervention characteristics and Inner setting
**Implementing PROGRESS**<br>• Ask them to explain how PROGRESS was put into place in their practice i.e. what happened first? – meeting/memo?<br>• Was there a plan for implementation? Were the goals and objectives clearly communicated? Who was involved in the planning?<br>• What type of training did they receive?<br>a) How useful was this?<br>• Were PROGRESS Champions appointed or did they volunteer for their role?<br>• Who did they go to with questions/issues, resource availability?<br>• Were other research studies or QI projects running at the same time – if yes, how was PROGRESS impacted?<br>• How did they become involved and what did they need to do to understand their role in PROGRESS and make it work?<br>• How did management support PROGRESS? What support did they receive from management?<br>• What would they do differently if they could start over again?	Coherence, Cognitive Participation, Collective Action, and Reflexive Monitoring	Outer and Inner setting and Process
**How are they delivering PROGRESS at their practice**<br>• Ask them to talk you through how they are delivering PROGRESS at their practice<br>o Overall process, the appointments/pre/follow up work<br>• Their involvement in delivering PROGRESS<br>• Are there differences in how individuals provide the service?<br>• What has worked well?<br>o Have they encountered any successes?<br>• What has not worked well?<br>o Have they encountered any challenges?	Collective action, reflexive monitoring	Inner setting, Individual characteristics, Process

o How have these been resolved?<br>• How does it compare to other services they provide?<br>• Have they had to change any existing processes/practices?<br>o Has it caused extra work?<br>• Have any costs been incurred through delivery of PROGRESS?<br>o Were costs considered when deciding to participate?<br>• Is PROGRESS being implemented as intended?		
**Impact on their practice**<br>• How is PROGRESS different from their practice's usual ways of prescribing medications?<br>• How does PROGRESS fit in with their other priorities and daily work? How do they juggle their workload?<br>• Is PROGRESS a normal part of their work?<br>• How does it fit with existing management care pathways for medication prescribing?	Coherence and collective action	Intervention characteristics, Inner settings and Process
**Perceived value in using PROGRESS for their work**<br>• Do they see value in using PROGRESS? Ask them to explain their answer<br>• If not? Explore why<br>• If no value in the present, what about in the future?<br>• What do they see as the potential benefits of PROGRESS?<br>a) For patients, for general practice, for health service generally<br>• What do they see as the potential drawbacks of PROGRESS?<br>a) For patients, for general practice, for health service generally	Coherence and Reflexive Monitoring	Intervention Characteristics, Individual characteristics and Process
**Feedback**<br>• Ask what type of feedback they have received about how implementation of PROGRESS is going<br>• How did they receive feedback?	Reflexive Monitoring	Inner setting and Process
**Communication across PCN and other sites**<br>• Ask if they meet with other people who work with PROGRESS across their PCN or other areas<br>• How do they feel communication is going with practices across other sites?<br>• If there is communication across sites, how useful do they find this for the work at their practice?	Cognitive Participation and Collective Action	Inner setting and Outer setting
**Communication within their general practice**<br>• Ask them to describe how they meet with colleagues to communicate about anything related to PROGRESS<br>• If they have meetings, how often do they meet? How useful do they find these meetings?<br>• How is the team able to adapt implementation of PROGRESS according to the feedback received?<br>• Ask how they communicate to each other about news, accomplishments, staff changes etc.	Collective Action and Reflexive Monitoring	Inner setting, Individual characteristics and Process
**How others perceived implementation of PROGRESS**<br>• Do they get a sense of how colleagues in their general practice feel about implementing PROGRESS (at the beginning and now)	Coherence	Inner setting and Characteristics of individuals

• Do they think there is a shared understanding among professionals and leadership in their general practice about the purpose and value of PROGRESS?<br>• How do they think it is received by patients?<br>a) Benefits/concerns voiced by patients<br>b) Are there different patient groups that are more or less receptive to participation in PROGRESS<br>Ask them to expand on their answer		
**How use of PROGRESS may have changed relationships with other colleagues**<br>• Ask them how using/implementing PROGRESS may have changed their relationships with other colleagues in their practice/PCN or with management<br>• Have they noticed a difference in relationships between colleagues / between colleagues and management since PROGRESS was put in place?	Cognitive Participation and Collective Action	Intervention characteristics and Process
**Views on future provision**<br>• Who is best placed to deliver PROGRESS?<br>• Is the current model sustainable in general practice?<br>○ Why/why not<br>○ What additional resources would be required?<br>○ How could current PROGRESS model be improved?	Cognitive Participation, Reflexive Monitoring	Intervention characteristics, Inner setting, Outer setting, Process
**Advice to other sites**<br>• Ask what advice they would give to future sites regarding implementation of PROGRESS?	Reflexive Monitoring	Process
**Is there anything else they would like to add about their experience with PROGRESS?**	N/A	N/A
