# Supplementary material for: Implementing pharmacogenetic-guided prescribing in general practice: a qualitative process evaluation
Source: Implement Sci Commun. 2026 Jun 26;7:149. doi: 10.1186/s43058-026-01005-x (PMC13471391; doi:10.1186/s43058-026-01005-x)
Supplement: Supplementary file 2 [file 43058_2026_1005_MOESM2_ESM.pdf]

PROGRESS_eval

Nvivo codes

Name
**Background**
interview length
practice characteristics
professional role (s)
**Communication**
communicating about project with staff
WhatsApp messages
communicating with other practices - acting as champion
presenting at conferences
**costs incurred**
**Future Provision - views**

Name
becomes an admin role to organise test
buccal swab instead of spit test
e-consent
give results to pts to own
incorporate into total triage pre-empt pts ahead of consultation
Integrating results into general practice electronic system
more pt education needed/awareness raising
more training needed
multidisciplinary input
need ability to update results as new testing available
Needs to be obvious who is eligible for test
Nurses in reviews could offer test for statins
Pharmacists' involvement
need to prove cost effectiveness

Name
requires sufficient resource
results available to all including secondary care
routine screening test
secondary care should offer test
self-testing
Should be offered to pts already on meds
simplify templates
Training needed on what results mean
whether primary care is best placed to offer test
**Impact on practice**
Can reduce workload in long run
have to make it fit in
How it fits with existing medication management pathways
How many pts recruited in

Name
likened to doing a blood or urine test
whether it changes prescribing practice
**Implementation process**
Delivering test in practice
barrier - communicating actions needed re contacting pt re results
barrier - competing priorities
barrier - excluding those on meds
barrier - filling out forms is clunky - research problem
barrier - no incentive to offer test
barrier - pop up fatigue
barrier - pt did test before consent - research problem
barrier - pts completing wrong sample etc.
barrier - tasks not being picked up
clinicians involved in offering test

Name
dependent on which staff prescribe which meds
people explain test in different ways
searching records for eligibility
how clinicians describe the test to pts
Maintaining continuity
Not part of routine care
overcoming teething issues
process of sending sample to lab
recruiting in wrong pts – research problem
research nurse consents pts
selecting pts to offer test
helps conversations re depression
Test done in practice
clinician unsure what happens to results

Name
when to prescribe medicines
delay prescribing till results returned
why some clinicians don't offer test
**process of organising test results**
barrier - having to log into GEN-O separate system
barrier - unclear on what test results given
interpreting or acting on test results
acts on other drugs not just one newly prescribed
local PI responsible for actioning test results
not possible if large numbers
maintaining continuity
**planning for implementation**
barrier - getting clinicians on board

Name
delegation of tasks
pharmacists organise results
got pharmacy involved after implementing
little evidence of planning for implementation
Not considered a big change - only a research study
slow to get started
Tools to aid implementation of pharmacogenetic test
barrier - pop up not working
Training from PROGRESS team SIV
training or informing staff
created video demonstration
Who was involved in implementation at practice
**Initial thoughts about PROGRESS**
co-designing project with PROGRESS team

Name
important to involve GPs in planning of study
Decision to adopt into practice
too complex to introduce across different sites
**Inner setting**
barrier - busyness of pirmary care impedes people offering test
barrier - not all staff trained in GCP
barrier - staff turnover
Doesn't see many new pts starting on statins
not everyone offers test
tailoring to local setting
whether other research impacts on PROGRESS implementation
management support
**lack of gaining feedback from others**
**making sure test is available to all**

Name
**miscellaneous - describing other project improvement campaigns**
**monitoring numbers**
**needs incentive**
**no public recognition for input**
**Outer setting**
Able to autonomously decide how to implement PROGRESS
constraints of research study
can be discriminating due to bureaucracy
No targets set for recruitment
**Perceived value**
Having results for future prescriptions
how it links to sustainability
perceived drawbacks
ethical dilemma - not acting on other results

Name
perception of others
barrier - difficult to produce enough saliva for test
Easy sell to pts
Pts who decline test
Results reflect real practice
**PROGRESS research team supportive**
**whether consent needed outside research study**
