# Supplementary material for: Implementing pharmacogenetic-guided prescribing in general practice: a qualitative process evaluation
Source: Implement Sci Commun. 2026 Jun 26;7:149. doi: 10.1186/s43058-026-01005-x (PMC13471391; doi:10.1186/s43058-026-01005-x)
Supplement: Supplementary file 3 [file 43058_2026_1005_MOESM3_ESM.pdf]

Theme	Quote
1. Perceived value influences prescribing practices	And I think if it was just in your arsenal, it would really alter the way that you communicate with this stuff… we're moving from paternalism into shared decision making, aren't we? Interview 13, GP (local PI), practice 07, phase 1 site.<br><br>I think it has definitely changed how I approach statins, I think, in terms of… the ones who are most resistant to it. Interview 24, GP (local PI) practices 15 & 20, phase 2 sites.<br><br>So, somebody will always [say] oh, [statins] they're terrible for this, that and the other... But there's that public perception of certain drug classes that, actually, has got out there and sometimes it's negative. So, if you can almost address [that]… [pharmacogenomic testing] does give that level of reassurance. Interview 11, Pharmacist (local PI), practice 06, phase 1 site.<br><br>But I think it was definitely useful for things like statins, very useful because we get lots of patients that do get side effects from statins, and this is a good way of reassuring them, by the way, you're a normal metabolizer for this, so it's unlikely that you're going to get any undue excess side effects with this. Interview 28, Pharmacist (local PI), practice 13, phase 2 site.<br><br>…to be honest, I'd mostly use it for SSRIs […] I guess from my personal experience, they're the ones that tend to be hit and miss and people chop and change on [SSRIs] a lot more, so to, like, try and negate some of that. Interview 4, GP trainee, practice 03, phase 1 site.<br><br>if you were scaling this up… antidepressants, anti-anxiety, that would be top of the list for me and statins…those are the top ones. Because usually I have said when I did the presentation [to clinicians at his practice], if there's anyone that you're thinking of being antidepressant or anti-anxiety and you want to start a medication, think of the PROGRESS trial because that would be the best suitable one. Interview 23, GP, practice 14, phase 2 site.<br><br>If they are on a statin, I want to find the right statin, if they are on an antidepressant, I want to find the right antidepressant. If they are on an opiate, I want them off it. Interview 15, GP (local PI), practice 08, phase 1 site.<br><br>I haven't tried even to do it with opioids, and we try not to prescribe them, even like a pain specialist, but we try not to

	prescribe them really. Interview 27, pharmacist (local PI), practice 18, phase 2 site.
2. Planning for implementation?	But what was quite good was they were still, sort of, codesigning the project over the time we were speaking to them…And so, we were involved maybe, you know, in helping shape that to a degree and helping advise [PROGRESS study team]. So, I suppose that helped to allay some fears, that you're an active participant in that. Interview 1, GP (local PI), practice 01, phase 1 site.<br><br>There was a hiccup in that one or two patients it wasn't activating [the GPAS online tool], once we'd put it on, and I'd participated in extra calls with – two or three extra calls – with EMIS, and with the PROGRESS team, and we identified a problem, which is actually quite good. […] we found a way round it, because we took time and I was happy to participate in these extra things. Interview 19, GP (local PI), practice 11, phase 2 site.<br><br>And I'm actually going to give them feedback on the first hundred patients at a teaching session in September…so, I think certainly, like, upskilling primary care clinicians in this is…was one goal. Interview 21, Pharmacist (local PI), practice 12, phase 2 site.<br><br>So, my plan is, you know the evaluation I was talking about … to present that at [next month's] afternoon closure, yeah, for half an hour or something…and I think that would increase awareness, and I'll go through the process again with all the GPs, and hopefully the clinical pharmacists will be there as well, and the nurses there. And this is what we're doing… and look at the result so far, isn't it great? Why don't you also participate? Interview 19, GP (local PI), practice 11, phase 2 site.<br><br>So, I got so far as to, you know, even recording my own short videos, apart from sharing all the training material information, recorded some two-minute knowledge nudges and things like that and shared them through WhatsApp and did internal coms on Teams, did some videos, some training, we did formal training sessions, you know, we've got a library of resources. Interview 17, GP (local PI), practice 09, phase 1 site.<br><br>It's been [Practice 01] that's actually been really supporting us, they're probably our nearest neighbours that are doing it and he's just sunshine, isn't he? Yeah, he's been eternally

	delightful, and they've been lovely to us. Interview 13, GP (local PI), practice 07, phase 1 site.
3. Delivering pharmacogenomics in practice: tailoring to local context	…it would certainly be on judgement, and it would be on that scenario and that particular patient […] And if I felt that it was necessary to start that medication first, then I would, I wouldn't hold off. Interview 18, Pharmacist (local PI), phase 1 site.<br><br>He said, if…and then he went on to say, do I need to wait for the results for this to start the [PPI] medication? And I said, well, actually, you've been without this [Aspirin] medication now for a number of years so really 10 more days isn't going to hurt anyone. So yeah, I said it's perfectly okay to wait for 10 days to get the results […] So yes, he was very happy with that. Interview 22, Pharmacist (local PI), practice 13, phase 2 site.<br><br>I always prescribe and then amend. So, I don't do both. And I think everyone in our practice does that. So, I think the decision was don't alter the [current prescribing practice]. Interview 9. GP, practice 04, phase 1 site.<br><br>And what I've learnt over time is, if you can, you're better waiting for the results. Only because sometimes the results come back, and they say they're a bit borderline […] there would have been a better option at the beginning, but you don't want to change someone's treatment now they've started it. Interview 1, GP (local PI), practice 01, phase 1 site.<br><br>I have tried to remove barriers so, for instance, [me and the senior GP partner] deal with all the results, so the other GPs don't really have to do anything… So, we have tried to make it as streamlined as possible to encourage people to recruit. Interview 8, GP (local PI), practice 05, phase 1 site.<br><br>Some of it comes down to, what's your attitude to your responsibility with cost-saving within the NHS? You've prescribed something and it's the incorrect thing, then it's a wasted prescription, wasted medication, it's wasted pharmacy times and stuff. Interview 3, GP (local PI), practice 03, phase 1 site.<br><br>I think… we're probably best suited for that and if there's any other complications…then we can obviously discuss with the GP about it. But actually, this should be a pharmacist domain. Interview 7, Pharmacist, (local PI), practice 04, phase 1 site.<br><br>As a pharmacist, we tend to probably have had a bit more training on metabolism elimination, all the pharmacokinetic

	things. Whereas I think the GPs are aware of it but it's not something they've ever, you know, learnt in detail. So yeah, I think it's definitely probably useful to be pharmacy led. Interview 21, Pharmacist (local PI), practice 12, phase 2 site.<br><br>…to make it more sustainable; I think you would need a system where the results were coming straight into the clinical system. I don't think…well, we can't cope with the additional log-ins. So, anything additional will…be a barrier. Interview 12, GP, practice 05, phase 1 site.<br><br>But if there was more local guidance: one, it would increase clinician confidence, they'll know where to look, because I think the CPIC guidelines aren't the easiest to navigate. And number two, if it was integrated into SystmOne in a way that wasn't a pop-up. I think that that would encourage clinicians to use it a little bit more too. Interview 25, Pharmacist (local PI), practice 17, phase 2 site.
4. Delivering pharmacogenomics in practice: barriers to use	A couple of the salaried [GPs] said, if I've got a late surgery, I cannot be expected to do this. And that was the end of that conversation. Interview 16, Research Nurse, practice 07, phase 1 site.<br><br>I suspect their hope was that we'd have achieved greater scale and I think that had been our hope too BUT, the NHS is on fire; that's quite a limiting factor. Interview 13, GP (local PI), practice 07, phase 1 site.<br><br>Different system of requesting, different system of resulting, it just feels different in every way. And it's not that different, but it's just those little barriers in a space where people are, like, urgh…, I can only handle one thing. Interview 17, GP (local PI), practice 09, phase 1 site.<br><br>So in my practice, and because we're newly setting up research, I'm the only one who is GCP trained and can actually do it, I'm the only person in the practice who is doing it. So, what I did was, at our clinical meetings which have our GPs, our nurses, our managers, I talked to them about the study, shared out the information. And then if they had anyone that they thought might be interested or they wanted to try it, they'd just refer them to me in my clinic, and then I'd go through the process. Interview 29, Pharmacist (local PI), practice 19, phase 2 site.<br><br>I created a predefined task template where, because I knew the GPs just wouldn't have the time to fill out the template to refer the patients to the study team, for, like, the consenting; and so the pharmacy team have been doing that with the GPs

	simply identifying, introducing the concept very briefly to the patient and asking the patient to collect a kit, and the pharmacy team will then be taking over. Interview 25, Pharmacist (local PI), practice 17, phase 2 site.<br><br>trying to convince other doctors especially to start talking about this, et cetera, and take the lead on it and do all the stuff, I think it's a bit much for them. But I do know that there are some other surgeries who are part of the PROGRESS trial where they do have everyone who does it, you know. So whatever works but, you know, the amount of busyness that we have, I don't…for the 10 minutes, 10 minutes that we've got to be with a patient, to add to do all that bit in is…you know, I think is a tall order. So that's why I'm the one that does it. Interview 23, GP (local PI), practice 14, phase 2 site.
